# Adenovirus-Associated Acute Interstitial Nephritis With Graft Survival and Novel Follow-Up Biopsy Findings Including Karyomegaly: A Case Series

**DOI:** 10.7759/cureus.38452

**Published:** 2023-05-02

**Authors:** Cullen M Lilley, Ewa Borys, Maria M Picken

**Affiliations:** 1 Department of Pathology, Loyola University Chicago Stritch School of Medicine, Maywood, USA; 2 Department of Pathology, Loyola University Medical Center, Maywood, USA

**Keywords:** immunosuppression complications, adenovirus, transplant infectious disease, infectious disease pathology, renal pathology

## Abstract

Adenoviral infections in post-transplant patients have been described in multiple organ systems, most classically the lung, liver, and alimentary tract. In the genitourinary tract, hemorrhagic cystitis is most frequently observed. Clinically apparent renal involvement with adenovirus is rare, and adenovirus-associated interstitial nephritis (AAIN) is an uncommon cause of renal allograft failure. Here, we present three cases of AAIN in patients who, after prompt diagnosis and treatment adjustment, experienced a return of allograft function. All patients were on standard triple therapy with tacrolimus levels within the target range at the time of biopsy. None of the patients had respiratory symptoms, and despite diarrhea, colon biopsies were negative. Only case one had positive adenovirus serology (IgG only) and case three had positive urine; two patients had leukopenia without neutropenia. Renal biopsies showed a characteristic granulomatous tubulocentric mixed lymphocytic and neutrophilic infiltrate. Adenovirus immunohistochemistry (IHC) showed strong staining in the tubular epithelium (nuclear and cytoplasmic) while staining for polyomavirus was negative. A follow-up biopsy two months after the diagnosis of AAIN in one patient revealed persistent cytopathic effects with negative adenoviral IHC staining while a biopsy at one year in another patient showed glomerular and tubulointerstitial scarring. AAIN is an uncommon but important etiology to consider in cases of acute renal allograft dysfunction. Although the presenting symptoms for AAIN are nonspecific, hematuria is frequently noted. Adenovirus IHC should be considered in cases with granulomatous inflammation associated with necrosis and mixed inflammatory infiltrate. As demonstrated in this single-institution case series, prompt diagnosis can result in the preservation of the renal allograft. Lasting cytopathic effects after adenoviral infection should also be considered in patients with a history, or potential history, of AAIN.

## Introduction

Apart from graft rejection, infections are important causes of early allograft dysfunction because of the immunosuppressed state of the host [1]. Infections of the renal allograft are commonly caused by polyomaviruses while adenoviruses have been identified as an uncommon cause of adenovirus allograft interstitial nephritis (AAIN) [1].

Adenoviruses are a diverse group of viruses belonging to the Adenoviridae family and have an equally diverse collection of virus-associated clinical syndromes [2]. In transplant recipients, adenovirus commonly affects the respiratory, alimentary, and urinary tracts [3]. In the urinary tract, adenoviruses typically cause hemorrhagic cystitis and can commonly affect the transitional epithelium of the ureters. In transplant recipients, adenovirus can affect the kidney parenchyma which can be difficult to diagnose on clinical grounds, but on kidney biopsy, there is characteristic tubulocentric granulomatous inflammation with visible viral cytopathic effect [1]. The diagnosis is typically confirmed with immunohistochemistry (IHC) against the group-specific hexon antigen which is meant to detect all clinically relevant adenoviral species and serotypes [2]. Though there are numerous species within this family, further molecular subclassification of adenoviruses is not common in clinical practice.

This article was previously presented as a poster presentation at the 2023 United States and Canadian Academy of Pathology (USCAP) Annual Scientific Meeting on March 15, 2023.

## Case presentation

Case identification

Cases of AAIN were identified and selected for review via a natural language search of the departmental archive. Over 15 years in a large academic center with approximately 220 kidney transplant biopsies performed annually, only three cases of AAIN were identified. Chart review and manual slide review were performed on identified cases. Pertinent information for each of the cases is organized in Table 1.

**Table 1:** Summary of patient cases. ng: nanogram; mL: milliliter; M: male; F: female; mo: months; IVIG: intravenous immunoglobulin

Case	Age/Sex	Presenting symptom	Time since transplant	Tacrolimus level at presentation (ng/mL)	Treatment
1	51/M	Fever, dysuria, organ pain, hematuria	1.5 mo	6.4	IVIG, cidofovir, ganciclovir
2	55/F	Fever, diarrhea, hematuria	22 mo	9.5	Decreased immunosuppression
3	61/F	Diarrhea, hematuria, dysuria	1.5 mo	8.5	IVIG

Case one

A 51-year-old male status post kidney transplantation for kidney failure secondary to hypertensive nephrosclerosis presented as a direct admit to the hospital 1.5 months post-transplantation; his immunosuppression consisted of prednisone, mycophenolic acid, cyclosporine, and tacrolimus. The major presenting symptoms were oliguria, hematuria, urgency, frequency, and kidney allograft pain. Imaging on admission confirmed proper ureteral stent placement and did not reveal any hydronephrosis. On admission, the patient was found to be febrile with an acute kidney injury with a creatinine of 2.65 mg/dL, up from a baseline of 1.9 mg/dL post-transplant. He was started on intravenous (IV) fluids, tamsulosin, and piperacillin/tazobactam antibiotic therapy. His tacrolimus level at presentation was found to be within the target range at 6.4 ng/mL. His urinalysis was significant for greater than 180 red blood cells (RBCs) per high-power field (HPF), 143 white blood cells (WBC)/HPF, and 2+ protein. Cultures of the urine and blood yielded no bacterial growth.

**Figure 1:**
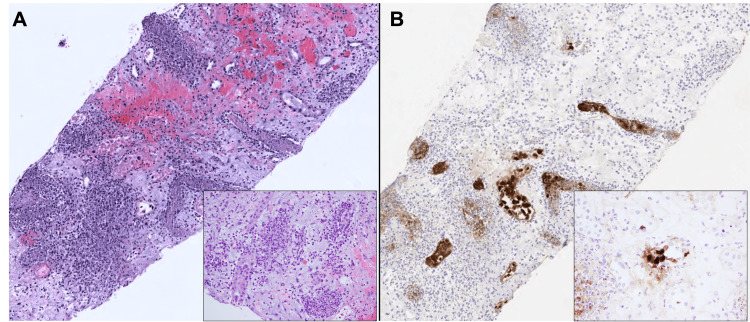
Representative photomicrograph of histologic findings in adenovirus-associated interstitial nephritis. Low-power photomicrographs of the findings on hematoxylin and eosin (A) and adenovirus immunohistochemistry (B) staining are depicted with higher-power images in the inset. Micrographs from patient one. Original magnification ×100, inset ×200.

A kidney biopsy was obtained on day three of hospitalization revealing dense tubulointerstitial inflammation with foci of necrosis. Acute cellular rejection was a major concern for the clinical team, and thymoglobulin was administered while awaiting kidney biopsy results. Kidney biopsy demonstrated a pattern of injury suspicious for infection of the renal allograft by adenovirus, and hence, adenoviral IHC was performed and found to be positive (Figure 1). Additional viral IHCs were performed including polyomaviruses, cytomegalovirus (CMV), and Epstein-Barr Virus (EBV) which were negative. Rejection workup including C4d immunofluorescence was negative. Additionally, the patient’s initial adenovirus serologies were found to be IgG-positive and IgM-negative (Table 2). Qualitative adenovirus and CMV real-time polymerase chain reaction (RT-PCR) of the blood was found to be negative; however, midstream urine adenovirus culture was positive.

**Table 2:** Summary of adenovirus laboratory findings. NA: not available; ELISA: enzyme-linked immunosorbent assay; IgG: immunoglobulin G; IgM: immunoglobulin M; mo: month; yr: year

Case	Adenovirus status in urine	Adenovirus status in serum/blood	Serology method	Serology during infection	Serology follow-up interval	Follow-up serology
1	Positive	Negative	ELISA	IgG+ IgM-	1 mo	IgG+ IgM+
2	NA	Positive	Complement fixation	Equivocal	1 yr	Positive
3	Positive	Positive	NA	NA	NA	NA

The patient was treated with five doses of IV immunoglobulin (IVIG) and a five-day course of cidofovir after receiving a single infusion of thymoglobulin. The cidofovir was stopped for concern of nephrotoxicity; however, after the termination of cidofovir, the patient’s hematuria persisted. The patient was given a cidofovir bladder irrigation and ganciclovir was started. After two days of the altered antiviral regimen, the patient’s creatinine began downtrending, and the patient’s symptoms improved. On discharge, repeat adenoviral serology revealed dually positive IgG and IgM antibodies. The patient’s allograft continued to function well. However, while a follow-up biopsy one year later revealed no rejection or suspicion of viral infection, it showed focal and segmental glomerulosclerosis and tubulointerstitial scarring which was not seen in the prior biopsies.

Case two

A 55-year-old female patient status post kidney transplantation for kidney failure secondary to end-stage renal disease likely due to the combined effect of diabetes and hypertension presented to the emergency department with fever, hematuria, dysuria, and diarrhea 22 months post-transplantation. In the emergency department, the patient was found to have obstructive uropathy due to a 3 mm stone. Urinalysis on admission was significant for greater than 180 RBCs/HPF, 17 WBCs/HPF, and 1+ protein. The patient was placed on empiric antibiotic treatment with piperacillin/tazobactam for suspected urinary tract infection (UTI) and metronidazole for empiric treatment of diarrhea. She was also treated with a nephrostomy tube placement for obstructive uropathy which improved renal function in the short term.

Urine from the nephrostomy tube was negative for bacterial growth and PCR of the urine was negative for CMV and BK virus. Although renal function improved in the days following the nephrostomy tube placement, the patient’s creatinine slowly began to rise. After adjustment of the nephrostomy tube and ureteral stone removal did not result in any improvement in renal function, a kidney biopsy was obtained on day 10 of hospitalization. The kidney biopsy demonstrated similar findings seen in case one and was confirmed by IHC to be AAIN. The complement fixation adenovirus serology assay was equivocal with a titer of 1:16 (Table 2). However, qualitative RT-PCR for adenovirus in the serum was also determined to be positive suggesting ongoing viremia. Serum qualitative RT-PCR of EBV, CMV, and BK were all negative. Further infectious studies including respiratory and stool panels were both pan-negative. Colonoscopy did not reveal any abnormalities, histologic analysis was unrevealing, and IHC against adenovirus, CMV, and polyomavirus were all negative in the colonic mucosa.

Before admission, the patient was on standard immunosuppressive therapy. Tacrolimus level at presentation was slightly elevated from the time since transplant at 9.5 ng/mL, and after equivocal antibody titers were detected, it was determined that decreasing immunosuppressive therapy would help the patient mount a sufficient response to the ongoing adenoviral infection. The patient was discharged after two days of altered immunosuppressive therapy with improved kidney function and followed up in the outpatient setting where her symptoms and kidney function returned to her baseline. Follow-up adenoviral serologies one year later were positive with a titer of 1:32 using the same method used during active infection.

Case three

A 61-year-old female patient status post kidney transplantation for kidney failure from an unknown etiology presented to the emergency department 1.5 months post-transplantation for diarrhea, burning with urination, and was found to have a ureteral stone. After an initial short hospital course where she was treated for a potential UTI with the placement of a ureteral stent that was subsequently removed after the passage of the stone, she returned to the hospital one day later with persistent diarrhea, fever, worsening kidney function, and leukopenia (WBC = 3.3 thousand cells/mL) without neutropenia.

The patient was on standard immunosuppression with a tacrolimus level within the target range at 8.5 ng/mL at presentation. Urinalysis taken in the emergency department was significant for greater than 180 RBCs/HPF, 91 WBCs/HPF, and 1+ protein. An infectious workup of the patient’s diarrhea, including a multiplex PCR and cryptosporidium antigen, that was pending on discharge was found to be pan-negative. On this admission, a colonoscopy was performed. A colon biopsy revealed normal colonic mucosa with no detection of CMV, adenovirus, or polyomavirus via IHC. On day three of hospitalization, a kidney biopsy was obtained, and a percutaneous nephrostomy tube was placed for potential obstructive uropathy.

**Figure 2:**
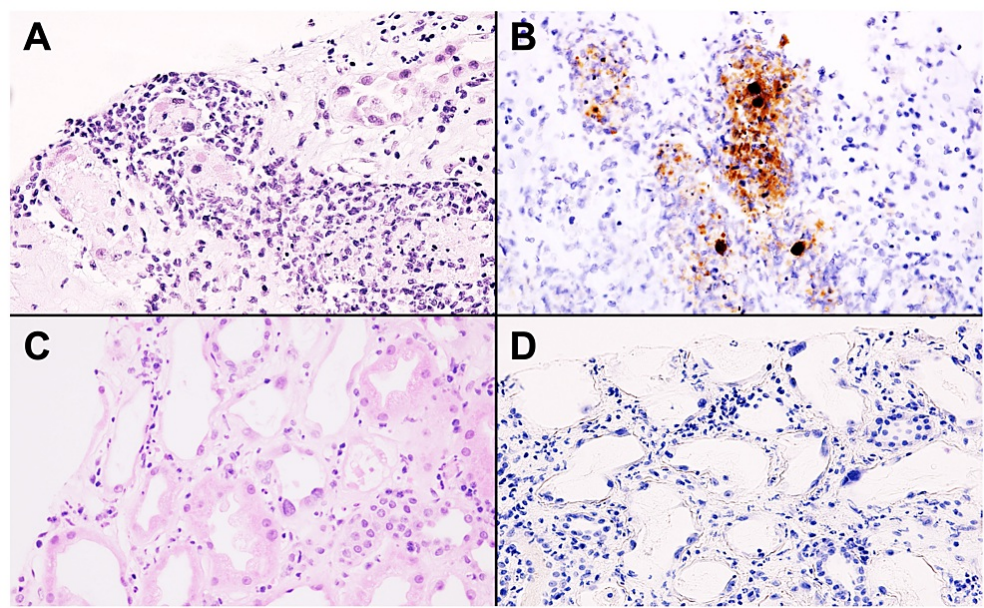
Representative micrographs from patient three during active infection and on follow-up biopsy. Representative sections of active infection (A, B) and post-infection persistent viral cytopathic effects (C, D) from patient three. Representative hematoxylin and eosin (A, C) and adenovirus immunohistochemistry (B, D) are shown. Original magnification ×400.

Kidney biopsy demonstrated interstitial inflammation patterns consistent with adenovirus interstitial nephritis confirmed by IHC which, when combined with the patient’s persistent fevers and worsening kidney function, necessitated the initiation of cidofovir. Adenovirus RT-PCR was positive in the serum and urine (Table 2). Cidofovir was discontinued after a single dose due to nephrotoxicity and a two-day course of IVIG was initiated. After treatment and surgical repair of the obstructive uropathy, the patient experienced an improvement in urinary symptoms, fever, diarrhea, and leukopenia. She was discharged with kidney function back to her post-transplant baseline. A follow-up kidney biopsy two months later revealed persistent cytopathic effects with karyomegaly and a smudgy nuclear appearance but a negative adenovirus IHC stain (Figure 2).

## Discussion

As illustrated by this single-institution case series and others in the extant literature, adenoviral infection of renal allografts is rare [3]. However, this infection can be associated with significant morbidity and ultimately graft failure.

A kidney biopsy is critical for the diagnosis of AAIN. The mixed inflammatory pattern seen in AAIN is distinct from the lymphocytic infiltrate seen in rejection and should prompt IHC analysis. All three cases in this series exhibited inflammatory responses similar to that reported in prior case reports and in a recent multi-institutional study. The three most common histologic patterns in AAIN include (1) granulomatous inflammation, (2) tubulocentric inflammation, and (3) acute tubular necrosis [1].

The follow-up kidney biopsy results from case three also highlight the importance of considering persistent cytopathic effects in patients with known or potential previous adenoviral allograft infection. This can be extremely challenging if a biopsy was not taken during the acute infection because the cytopathic effects identified in this case closely resembled that of karyomegalic interstitial nephritis [4]. Cidofovir toxicity as a potential cause of karyomegaly is unlikely in this patient who received only a single dose of the drug [5]. The extent of persistent viral cytopathic effects has not been determined in the extant literature and the case presented in this series is, to our knowledge, the first to report this finding. A follow-up biopsy of case one obtained one year later demonstrated glomerular and tubulointerstitial scarring. However, the short and long(er)-term sequelae of AAIN are largely unknown. All three cases highlight the value of a kidney biopsy in diagnosing allograft infections, including those which are uncommon such as AAIN.

Similar to prior reports, both cases, one and two, illustrate the difficulties with the interpretation of serologic tests to determine the stage of adenoviral infection in the transplant recipient [6]. In fact, as reported previously, serologies alone should not be used to diagnose acute infection [7-10].

Importantly, other laboratory tests to detect adenoviruses such as RT-PCR and antigen detection are also not reliable in cases of AAIN, as illustrated by the varied results exhibited by patients in this series. Viremia, though common in AAIN, is not seen in all cases of AAIN, and should not serve as the only means of diagnosing this condition [3]. A similar consideration applies to the detection of adenovirus in the urine. Importantly, in none of the cases identified in this series, we were able to detect adenovirus through RT-PCR or multi-pathogen PCR panels in either the gastrointestinal or the respiratory tract.

While hemorrhagic cystitis has been reported to be frequently associated with AAIN, clinical symptoms exhibited by these patients are not uniform. Thus, in the second patient in this case series, kidney biopsy was delayed for 10 days due to clinical presentation suggesting bacterial infection with obstruction as a possible cause of allograft function deterioration.

Interestingly, all three cases identified in this case series also coincidentally involved ureteral foreign bodies (stones, stents) or ureteral manipulation shortly before the identification of AAIN. Clinically relevant adenoviruses are known to infect a multitude of organs in the body, but the B-type adenoviruses, in particular, have the propensity to infect the urinary tract and cause hemorrhagic cystitis, especially in immunosuppressed patients who recently received a solid organ transplant [2,3,11]. While the exact mechanism by which adenoviruses seed tubular epithelial cells and cause AAIN is unknown, it is feasible that the presence of a ureteral foreign body or external ureteral manipulation may raise the likelihood of ascending infection to the allograft from the bladder rather than hematologic spread from a primary gastrointestinal or respiratory infection. This ascending infection hypothesis is also supported by the high proportion of such cases in larger (n = 11) retrospective cohort studies of patients with AAIN [1]. This theory is also supported by the inability to detect adenovirus in the respiratory or gastrointestinal tract via PCR-based methods.

Donor-derived adenovirus infection was also reported in two patients who received kidneys from the same donor and developed AAIN within <1.5 months post-transplantation [12]. Interestingly, two of our patients developed AAIN within a short post-transplantation period (1.5 months) but we have no information about the recipients of the other kidney(s) from the same donor.

## Conclusions

Adenovirus infection is common in patients on immunosuppressive therapy in the post-transplant period. However, adenoviral infection is an uncommon but important cause of interstitial nephritis in the setting of the post-transplantation immunosuppressed state. Though there are laboratory tests to assess adenoviral viremia as well as antibody response to prior infection, in cases of allograft dysfunction of unknown etiology, it is important to obtain a kidney biopsy to assess for common causes of allograft dysfunction as well as viral cytopathic effects and presence of viral antigens through IHC analysis. Based on this single-institution case series, early diagnosis and treatment alteration were key to graft survival. Additionally, the final case in this series highlights the importance of considering prior AAIN in patient biopsies with viral cytopathic effects and negative antigen reactivity on IHC analysis, a phenomenon that has, until this paper, not been reported in the extant literature.
